# Towards navigation in endoscopic kidney surgery based on preoperative imaging

**DOI:** 10.1049/htl2.12059

**Published:** 2023-12-13

**Authors:** Ayberk Acar, Daiwei Lu, Yifan Wu, Ipek Oguz, Nicholas Kavoussi, Jie Ying Wu

**Affiliations:** ^1^ Department of Computer Science Vanderbilt University Nashville Tennessee USA; ^2^ Department of Urology Vanderbilt University Medical Center Nashville Tennessee USA; ^3^ Present address: Department of Computer Science Vanderbilt University Nashville Tennessee USA

**Keywords:** endoscopes, image reconstruction, kidney, surgery

## Abstract

Endoscopic renal surgeries have high re‐operation rates, particularly for lower volume surgeons. Due to the limited field and depth of view of current endoscopes, mentally mapping preoperative computed tomography (CT) images of patient anatomy to the surgical field is challenging. The inability to completely navigate the intrarenal collecting system leads to missed kidney stones and tumors, subsequently raising recurrence rates. A guidance system is proposed to estimate the endoscope positions within the CT to reduce re‐operation rates. A Structure from Motion algorithm is used to reconstruct the kidney collecting system from the endoscope videos. In addition, the kidney collecting system is segmented from CT scans using 3D U‐Net to create a 3D model. The two collecting system representations can then be registered to provide information on the relative endoscope position. Correct reconstruction and localization of intrarenal anatomy and endoscope position is demonstrated. Furthermore, a 3D map is created supported by the RGB endoscope images to reduce the burden of mental mapping during surgery. The proposed reconstruction pipeline has been validated for guidance. It can reduce the mental burden for surgeons and is a step towards the long‐term goal of reducing re‐operation rates in kidney stone surgery.

## Graphical abstract

Endoscopic renal surgeries have high re‐operation rates, particularly for lower volume surgeons due to visualization and mental mapping challenges. In this work, we create 3D reconstructions from endoscopic images and register them to automatically segmented preoperative CT scans to create patient‐specific 3D maps. These maps can be used as a feedback mechanism and they provide frame‐based localization in CT scans.

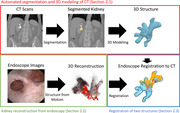

## 1 INTRODUCTION

Endoscopic surgery is the standard of care for the treatment of diseases of the renal collecting system such as stones and tumors. By using small endoscopes, urologists can perform natural orifice procedures, minimizing morbidity for patients. However, current endoscopes used in urological surgery have a limited depth and field of view, leading to impaired visibility and complicating accurate localization within the intrarenal anatomy [1, 2]. Furthermore, factors like organic debris and blood clots (which increase throughout surgery) further impact visibility. Thus, completely visualizing the collecting system anatomy during surgery often depends on the surgeon's ability to transform and mentally register a series of 2D preoperative axial computerized tomography (CT) images of the patient's anatomy to the endoscopic surgical field. Since mental mapping relies on hand‐eye coordination, memory, and spatial reasoning based on preoperative imaging in a branched intrarenal collecting system, it is inherently imprecise and dependent on surgeon experience. This can lead to poor coverage of the kidney during stone identification phase of the kidney stone surgery. Residual stones or tumors can be left behind by the surgeon in unexplored branches of the collecting system, ultimately leading to a need for re‐operation.

**Novel contribution of our paper**. We propose a pipeline to integrate preoperative CT information and endoscopic videos. This is a step towards providing intraoperative guidance based on 3D models from pre‐operative imaging. Our algorithm can show the endoscope pose within a patient‐specific 3D map, supported with RGB endoscopic images within the collecting system. This can mitigate the effects of any in vivo endoscopic visualization difficulties, enhancing intraoperative navigation during endoscopic surgery. This study presents a novel application for the combination of methods and their evaluation. To the extent of our knowledge, this is the first study to evaluate, create, and use complete reconstructions of the kidney collecting system.

### 1.1 Previous work in endoscope image localization

**FIGURE 1:**
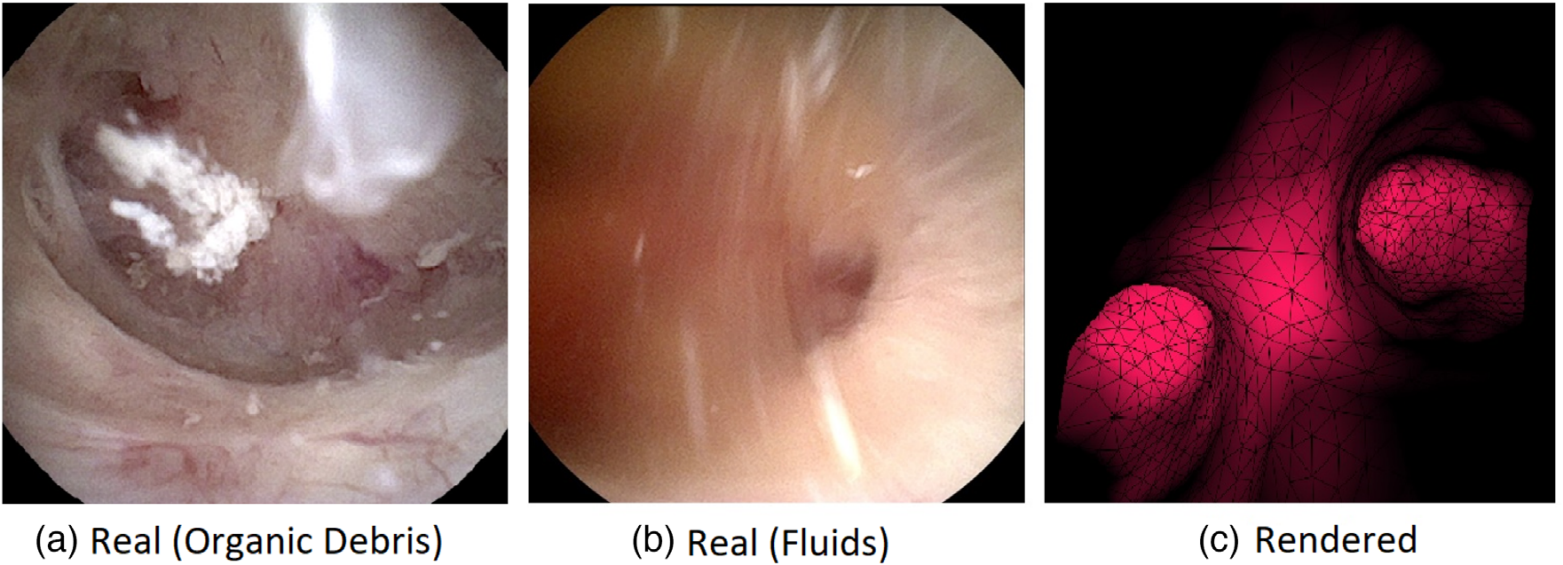
Examples of real and rendered endoscope video frames. Organic debris and the flow of the liquids in real endoscope videos hinder the reconstruction.

Previous works related to endoscope localization during surgery mostly rely on image matching between real endoscope frames and rendered images. Otake et al. propose a pipeline that estimates the camera position by calculating the similarity between the rendered and real endoscopic images in sinus surgeries [3]. Similar approaches are used in bronchoscopy [4, 5] and in other sinus procedures [6]. While these methods may give a fair estimation of the endoscope position in each frame for air‐way‐based procedures, visual challenges, such as fluid motion and organic debris seen in Figure 1, in endoscopic kidney surgeries lead to a greater difference between rendered and real images. When the similarity is lower between the rendered images and surgery videos, they cannot be matched accurately. These problems also decrease the accuracy of depth estimations and hinder the use of depth‐based registrations. Han et al. suggest a Structure from Motion (SfM) 3D reconstruction‐based method to register endoscope images to CT scans [7] but the reconstruction is limited to only small portions of the structure and a complete mapping is not created from endoscopic images. Therefore, it requires a multi‐branch structure, where registration and localization are limited to the branch openings.

### 1.2 Previous work in structure from motion

SfM algorithms are widely used for the 3D reconstruction of internal structures. These algorithms detect the feature points in each frame and find the matches with others. Since they follow distinct features through motion, it is possible to obtain an estimation of the camera position throughout the complete trajectory using the different viewpoints on the tracked features. With advances in feature extraction and matching algorithms, the capabilities of SfM are increased. Bianco et al. compare the SfM algorithms on a synthetic dataset containing real objects such as bicycles, cars, statues etc. and conclude that COLMAP [8] performs best in many use cases among widely used algorithms [9]. Widya et al. implement reconstruction of the stomach using COLMAP SfM [10]. Results were limited with raw endoscope images but the use of indigo carmine (IC) dye improved their reconstruction. There is no similar dye used in kidney stone surgery. In follow‐up work, results are improved using virtual IC dye, and localization is achieved using the projection of the camera positions on the reconstructed meshes [11]. Nevertheless, a lack of IC dye ground truth images and translation to the CT domain limits the usability of this algorithm for our task.

### 1.3 Previous work in improving feature detection

Endoscopic images lack distinct features such as corners and keypoints that can be observed in everyday objects. This limits the usability of the algorithms that use image features in endoscopic images (i.e. SfM, SLAM) but results can be improved using additions to the structure such as IC dye and relatively easy applications of image processing. With the presence of liquid flow and organic debris (Figure 1a,b) and without visually identifiable landmarks and supporting factors, feature detection in kidney endoscope images becomes a different challenge than other internal structures. While default reconstruction on COLMAP uses scale‐invariant feature transform (SIFT) [12] to detect and match the features, it fails to create a complete reconstruction of the kidney collecting system due to the mentioned difficulties. Pixel‐Perfect SfM [13] refines the keypoint, uses bundle adjustments, and allows easier implementation of SuperPoint feature extraction [14] and SuperGlue feature matching [15] by using the Hierarchical Localization Toolbox [16]. In their comparison, SuperPoint and SuperGlue algorithms perform best in the accuracy and completeness of 3D reconstruction.

In this paper, we propose a pipeline for determining the pose of the endoscope within a 3D patient‐specific model based solely on the endoscope images. This is the first step towards creating an intraoperative tool to reduce the mental burden for clinicians to mentally match the 3D pre‐operative CT scan to the intraoperative endoscope image. It can help achieve more thorough coverage of the kidney by indicating which parts have already been explored on the 3D model and thus reduce reoperation rates in kidney stone surgery.

## 2 METHODS AND RESULTS

**FIGURE 2:**
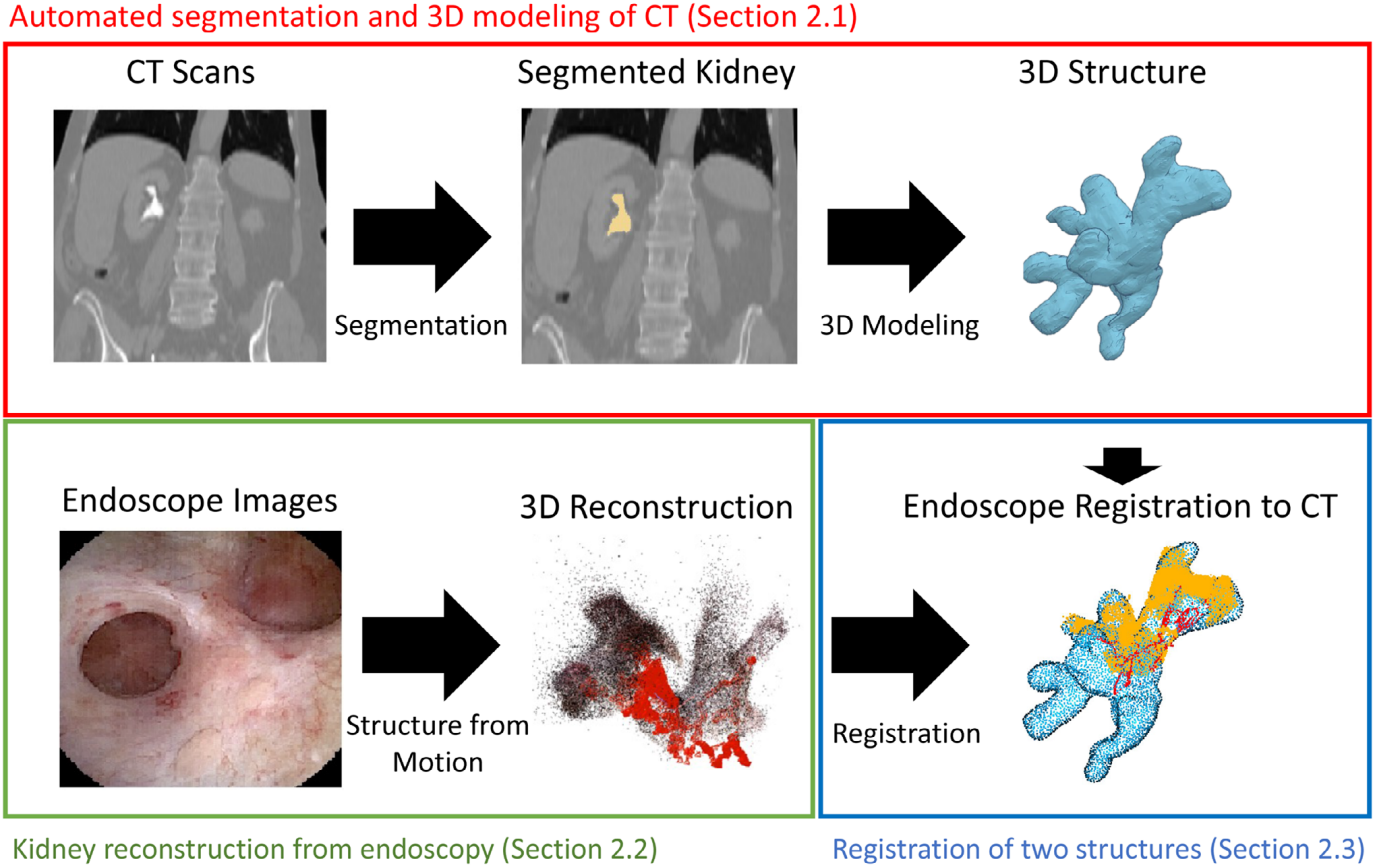
General workflow of the algorithm. We use an automated segmentation algorithm to create a 3D model of the kidney collecting system, while the SfM algorithm creates a 3D reconstruction from endoscope videos. Kidney reconstruction is registered to the 3D structure generated by CT, the blue point cloud represents sampled CT structure and the yellow point cloud represents the volume covered in the endoscope video.

The overall goal of our work is to find the position of the endoscope within the CT scans. As the pipeline has several components that are considered separately, we combine our method and results section and instead separate it by component. We first automatically segment the CT scan using 3D U‐Net [17] to create a patient‐specific 3D kidney collecting system model. We then reconstruct a 3D model from the endoscope images using SfM. The SfM algorithm estimates the endoscope trajectory in addition to reconstructing the collecting system model. After segmentation and reconstruction, we use 3D registration to align the reconstructed model from the endoscope to the segmented model from the CT scan (Figure 2). This allows the surgeon to see where the endoscope is located within the collecting system, as well as what segments of the kidney have been explored so far.

### 2.1 Automated segmentation and 3D modeling of collecting system

In the first stage of our pipeline, we segment the collecting system from CT scans and use 3D modeling to create the registration baseline. We first segment the entire kidney from CT scans using a 3D U‐Net model, which is widely used in many different application areas, including medical image segmentation [18, 19]. To decrease the noise arising from the relatively small size of the collecting system, we extract the target structure as a post‐processing step.

Our dataset contains 17 delayed‐phase CT scans with a combination of healthy visitors and patients diagnosed with upper tract urothelial carcinoma and kidney stones. A graduate student labeled the scans with the assistance of a surgeon, using ITK‐SNAP [20]. First, we scale the scans to 256×256×256 voxel size. To increase the sample size, we applied augmentations such as random cropping, intensity shifts, and affine transformations.

In model input, we use 128×128×128 patches and train the model using MONAI [21]. We use sliding window inference for prediction with a 0.5 overlap ratio and train the model using 6‐fold cross‐validation, with 11 train 3 validation 3 testing samples at each fold. For collecting system extraction, we perform dilation with a 5×9×9 kernel and a three‐class Otsu thresholding [22] on the segmented kidney. We select the area with the highest intensity for the collecting system, as a result of the accumulation of contrast material in delayed‐phase CT. For final results, we acquired average Dice scores of 0.842±0.139 for the whole kidney and 0.853±0.084 for the collecting system.

For the 3D modeling of the system, we create a 3D mesh from segmented CT scans using 3D Slicer [23] model maker, which is an implementation of marching cubes [24]. As the last post‐processing step, we dilate the structure by adding the normal values to the coordinates of each vertex using Meshlab [25], to account for the expanded kidney during the operation due to irrigation.

### 2.2 3D reconstruction

3D reconstruction from endoscopic images is inherently difficult as the visualized features are not always distinct. The goal of this section is to explore whether the fundamental structure in kidneys (i.e. infundibula draining calyces and renal papillae to the renal pelvis), provides sufficient features for existing reconstruction algorithms. In addition, we target pathological kidneys with stones, which may provide additional features for reconstruction. We compare the reconstruction performances of the widely used regular COLMAP [8] pipeline with the Pixel‐Perfect SfM [13] framework. In our comparisons, we used COLMAP with SIFT and Pixel‐Perfect SfM with SuperPoint [14] and SuperGlue [15]. We also evaluated the performance of SuperPoint and Superglue without the Pixel‐Perfect refinement. To test the success of reconstruction and registration without considering the challenges endoscope videos pose for feature detection, (Figure 1) first, we applied the 3D modeling stages explained in previous section to a manually segmented CT scan. Then, we rendered a camera trajectory on the mesh of the kidney using the 3D Slicer endoscope module.

The rendered endoscope trajectory avoids confounding factors for feature detection such as liquids or floating debris. We evaluated the algorithms both qualitatively and quantitatively in three real endoscope videos and two rendered ones. Table 1 shows the total number of frames extracted from the videos, those successfully used in the reconstruction, and the number of reconstructed points. Note that while the algorithms may produce more than one reconstruction, we select the one with the most images to use in comparisons.

**TABLE 1:** Comparison of Structure from Motion algorithms. Each column indicates a different input video. With an increasing number of images, more information coming from different frames is used which results in wider coverage. Reconstruction percentage gives the ratio of successfully used frames in reconstruction to total frames. More points also indicate more features found and matched, leading to more details. Bold values shows the best result for the corresponding input video.

	Rend. 1	Rend. 2	Real 1	Real 2	Real 3
Total images	454	542	1253	1454	1966
**COLMAP**					
Reconstructed points	6117	3558	**45800**	119542	**92709**
Reconstructed images	124	75	414	414	301
Reconstruction percentage	27.31%	13.84%	33.04%	28.47%	15.31%
**SuperPoint + SuperGlue**					
Reconstructed points	32679	**52681**	6740	172552	11845
Reconstructed images	**241**	344	**573**	1402	**565**
Reconstruction percentage	**53.08%**	63.47%	**45.73%**	96.42%	**28.74%**
**Pixel‐Perfect**					
Reconstructed points	**32924**	52508	30	**175293**	7315
Reconstructed images	**241**	**347**	12	**1406**	410
Reconstruction percentage	**53.08%**	**64.02%**	0.96%	**96.70%**	20.85%

**FIGURE 3:**
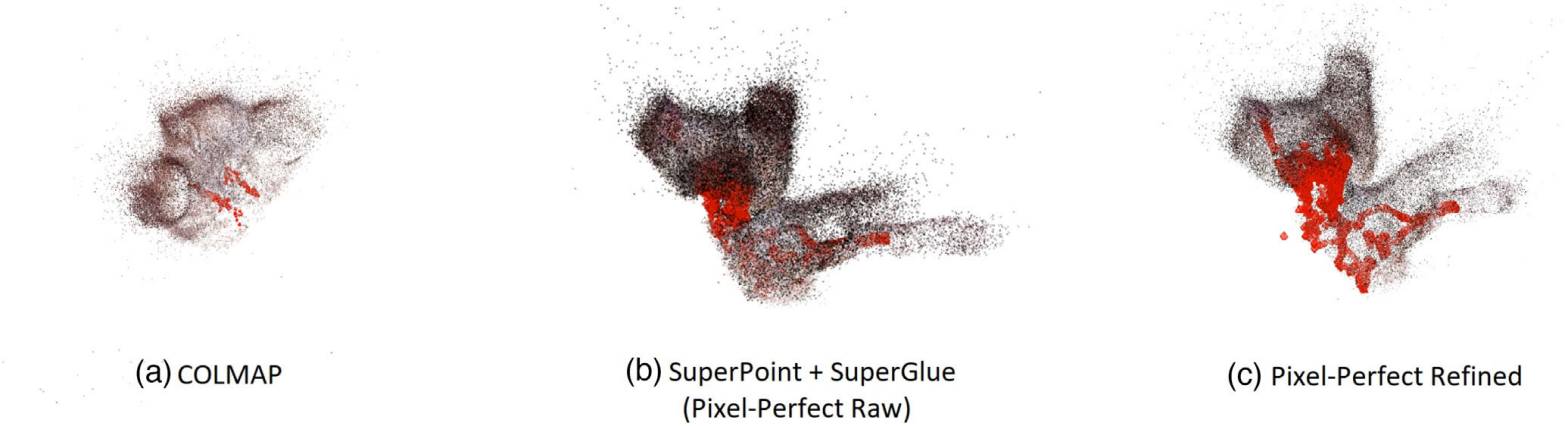
Reconstruction results from different algorithms. COLMAP manages to create a partial reconstruction of the images while other algorithms reconstruct all the branches covered in the endoscope video and have a higher coverage.

For our task, acquiring a complete overall reconstruction is more important than having a detailed reconstruction of a smaller area. In general, methods using SuperPoint and SuperGlue give better results on the reconstruction matching our aims. We observe that Figures 3b and 3c created more complete reconstructions of the collecting system structure whereas Figure 3a only reconstructed a small portion.

**FIGURE 4:**
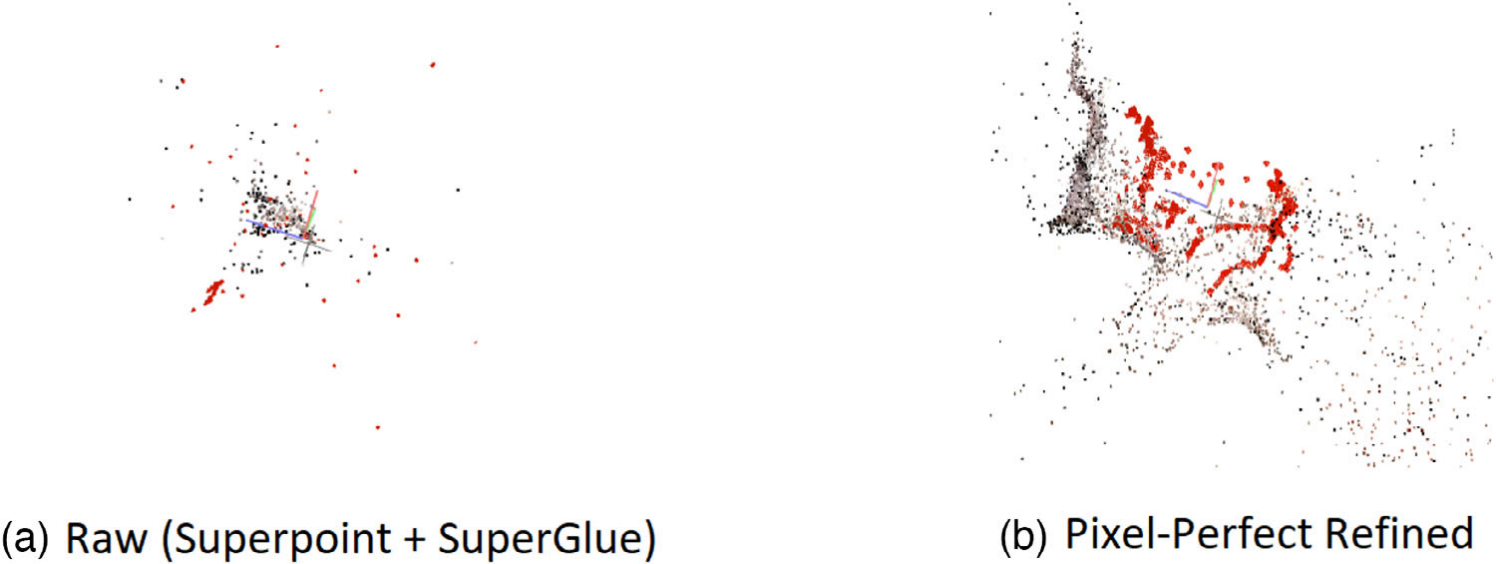
SuperPoint + SuperGlue failure case. While raw version (a) includes more points and images, fails to create a useful structure for registration. Refined version (b) gives a better outline of the kidney for camera position localization.

Refinement features of Pixel‐Perfect may improve performance and decrease noise and also eliminate weaker reconstructions (e.g. Real 1 column in Table 1). Even though SuperPoint and SuperGlue have higher reconstruction rates for some cases, the resulting point cloud may not contain enough structure to be useful for the rest of the algorithm (Figure 4). Since denoising is a useful feature in registration and weaker reconstruction remains a limitation for the whole method, we decided to proceed with the Pixel‐Perfect algorithm. However, SuperPoint + SuperGlue pipeline without refinement can be used as an alternative when it finds a non‐trivial reconstruction.

For the analysis of the complete pipeline, after institutional review board approval, we collected a matching endoscope video and CT scan (dimensions 512×512×82 voxels and voxel spacing $0.76\times 0.76\times 5\;{\mathrm{mm}}^{3}$) set for a patient undergoing kidney stone surgery. We test the proposed method with a real endoscope video of 97 seconds and 30 FPS. After extracting the video frames and downsampling them by 2 for faster processing, we acquire 1253 endoscopic images. We create the reconstructions using the Pixel‐Perfect SfM pipeline with SuperGlue and SuperPoint feature extractor and matching algorithms.

**FIGURE 5:**
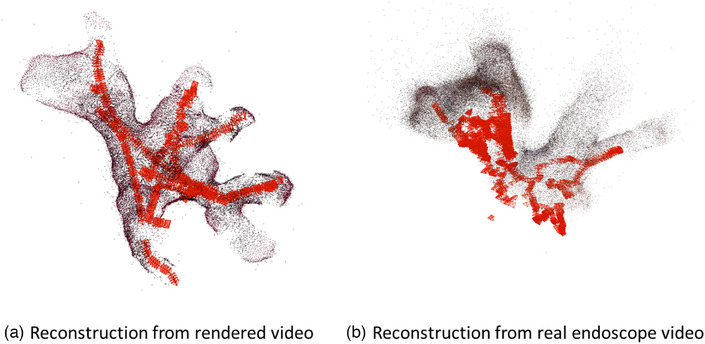
Reconstruction results for rendered and real images. Red marks represent the camera trajectory and frames used in the reconstruction of the particular area.

In order to compare and test the reconstruction capabilities under ideal conditions, we apply another reconstruction to a virtual endoscope video since the reconstruction of rendered images is easier without liquid and organic debris, and with meshes on the structure for easier feature detection. We use manually segmented and undilated CT for the rendered endoscopy tests. After the extraction of frames and sampling, we acquired 454 virtual endoscopy images. Since the rendered endoscope video covers the whole structure and contains more detectable features with the presence of meshes and without real‐life challenges, a better point cloud representing the complete structure is achieved. For the real endoscopy video, all the branches visible in the video are reconstructed correctly as well (Figure 5).

### 2.3 Registration and localization

**FIGURE 6:**
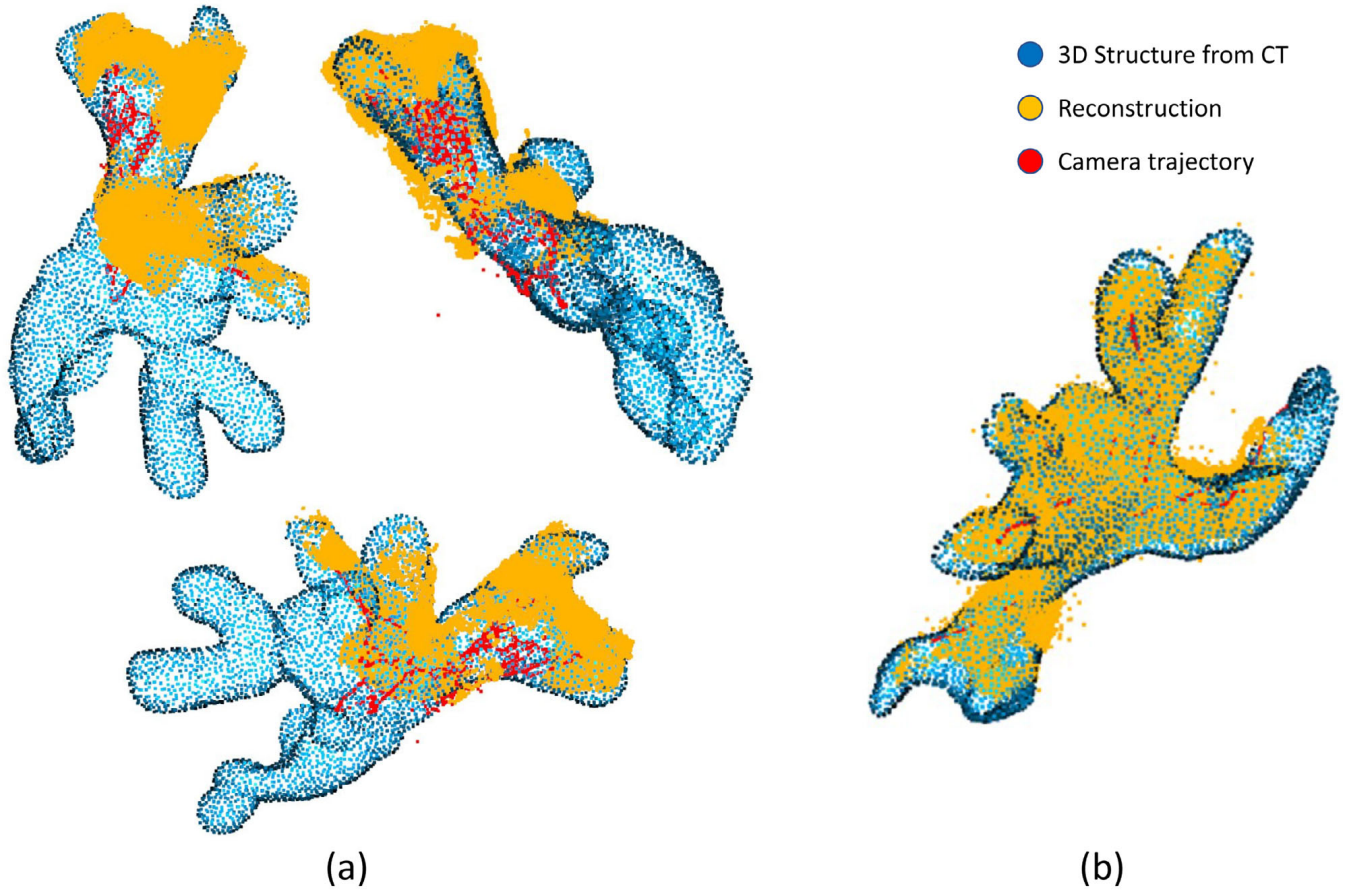
(a) Final results of the algorithm for real endoscope images from different angles. (b) Results for the rendered videos. The blue point cloud shows the CT model, the yellow point cloud shows the reconstruction, and the red marks indicate the camera path. Each red dot represents a frame used in the reconstruction.

To localize the endoscope pose within the CT and create the final 3D map, we next register the point cloud reconstructed from SfM to the CT domain using Open3D [26]. We start the registration procedure with voxelization of the 3D structure achieved from CT scans and downsample both structures as preprocessing steps. Then, we estimate the normals of input point clouds and calculate Fast Point Feature Histograms (FPFH) [27] for each point. Since the reconstruction may end up with outlier points that disturb the flow of the registration, we apply a radial nearest neighbor filter. With the use of this filter, we only preserve points that have a neighbor within a specified radius and discard the rest. For global registration, we first tried applying RANSAC [28] with the steps explained in Open3D documentation 1, which refers to Choi et al. for the parameters [29]. However, the global registration phase requires a good initialization on the scales. This may be unavailable due to deformations of the kidney as it is filled with fluid during the operation. As a result, while this registration works well for rendered images and structures, we observed instability and changing results for the real reconstructions. Also taking the accumulating errors from segmentation and reconstruction into account, we manually tune the scales of the point clouds by using the dimensions of two point clouds as a reference and select corresponding reference points from both structures using a simple user interface. We use the manually selected correspondences for initial point‐to‐point registration, replacing RANSAC for global registration in real endoscope video tests. While the general dimensions are useful in estimation, they cannot be directly used in the case of partial reconstructions, and manual intervention is needed. As a final step to acquire the transformation, we apply Iterative Closest Point (ICP) [30] for refinement. We use the transformation we acquire on the point cloud of the reconstruction and the camera positions given by SfM. Using the camera pose estimation feature of the SfM, we achieve an endoscope position estimation for each frame. On the resulting registration, coverage of endoscope videos, endoscope tip positions for each endoscope frame used in the reconstructions, and the general path of the endoscope can be easily observed (Figure 6).

Analysis of the registration is limited since the acquirement of real‐time tip position in current practice is not possible. The accuracy of the registration can be inspected visually and using the metrics of the registration algorithm. Table 2 shows the root mean square error (RMSE) values and the ratio of the points considered as inliers by the Open3D algorithm to all points in the reconstruction. We observe sub‐millimeter errors in the registration of both reconstructions, while the inlier to total points ratio is lower in real endoscope reconstruction. Values and visual representation of the rendered video could be considered as reference points due to the lack of ground truth, while the differences may come from the deformation of the kidney.

**TABLE 2:** Quantitative results for registration using Open3D. Inlier RMSE shows the Root Mean Square Error calculated from the points considered as inliers and Inlier Points/Total Points shows the ratio of these points to all points in the reconstruction point cloud.

	Inlier RMSE	Inlier/total points
Rendered video	0.4335 mm	28305/52508
Real endoscopy	0.7052 mm	70988/175293

The runtime of the complete algorithm is around 8 hours for a workstation with Intel Xeon Processor E5‐1630 v4 and NVIDIA Titan V running on Ubuntu 20.04 when the images are used in maximum quality and the default parameters are selected for the reconstruction. We note that there is a trade‐off between the reconstruction quality and duration, depending on the system used and the required quality this duration may vary.

## 3 DISCUSSION

We present a 3D map that can aid mental mapping and act as a reference intra‐operatively during endoscopic kidney surgery. Our qualitative result demonstrates that our reconstruction has good coverage of the kidney collecting system and our quantitative results show that the reconstruction is sufficient for accurate registration to the preoperative CT. In current practice, visualization of the anatomy highly depends on the surgeon's skill. However, as tumor/stone or collecting system complexity increases, mentally registering preoperative kidney tumor/stone information to the endoscopic anatomy becomes difficult, causing a surgeon to incompletely treat these pathologies [31]. Endoscope images present a very limited field of view and depth of field (10 mm and 6 mm on average, respectively). By registering endoscopic surgical video to segmented, preoperative CT images, we create a navigational system that notifies a surgeon of the endoscope and tumor/stone position within the collecting system to facilitate treatment. Furthermore, in patients undergoing endoscopic surveillance procedures for kidney tumors, image matching could be used to allow for localization of previous sites of tumors and evaluate for recurrence. The patient‐specific map, supported by endoscope RGB images can be used to create patient‐specific surgical plans. Such technology could also be used to aid in future autonomous surgical systems.

Despite the potential of our technology, there are several limitations. First, reconstruction and map generation capabilities are highly correlated with video qualities. Fluid motion and organic debris also introduce difficulties in accurate reconstruction for endoscopic renal surgery. We may get false feature matches for stones and debris, which are not in the mesh extracted from CT. We also observe deformation in the reconstructed structure such as contractions in the volume due to fast camera motion. While any deformation makes the registration process harder, we use partial registrations to circumvent inexact matches. We break the reconstruction point cloud into pieces to overcome contractions and extensions in the branches while preserving the landmark points such as branch openings. Additionally, we apply dilation to the 3D model obtained from the CT scans to account for the volume changes due to irrigation.

Although we obtain sub‐millimeter registration errors, evaluation is limited since ground truth information for camera positions is not available in current practice. We plan to extend our testing with realistic phantoms and ex‐vivo studies, where we can have better control over camera trajectories and collect ground truth data for camera positions. Based on our current evaluation, high accuracy registration is not needed for having a general idea about camera trajectory and the assessment of major calyces coverage. However, higher registration accuracy would enable a more detailed analysis of minor calyces. Future work can focus on using anatomical landmarks or known camera positions such as start and end points for initialization of registration and increased accuracy.

While our pipeline currently does not support real‐time processing, the acquired patient‐specific map can be used as a guidance tool in future surgeries. It can also be used for evaluation of operation success as indicated by the percentage of the kidney collecting visualized during the surgery. It can provide real‐time localization and has distinct landmarks according to the patient anatomy. Future work may include applying a filter to reduce the noise in the images or training feature extractors on kidney endoscope image‐specific landmarks to reconstruct 3D volume from lower‐quality videos. Furthermore, we are working on simultaneous localization and mapping (SLAM) algorithms and improvements in the registration method such as using more advanced algorithms or automatic scaling to make this pipeline completely automated and real‐time.

## 4 CONCLUSION

In this work, we combine the information from CT scans and endoscope videos. We create a 3D reconstruction of the kidney collecting system using SfM and register it to the 3D structure created with the automated segmentation of kidney CT scans. Furthermore, we localize the endoscope pose in the CT domain. We create a 3D mapping of the kidney supported by the visual information from the endoscope images, which can be used as landmarks for any future surgeries. Finally, we represent the surgeon's coverage of the branching structures inside the kidney during the endoscopy procedure. This work is a step towards a 3D guidance system for endoscopic kidney surgery. By registering the endoscope video to the CT image, we reduce the mental burden for clinicians to maintain a mental 3D anatomical model. This has the potential to improve navigation during endoscopic kidney surgery and reduce re‐operation rates.

## AUTHOR CONTRIBUTIONS

**Ayberk Acar**: Conceptualization; investigation; methodology; project administration; software; visualization; writing—original draft; writing—review and editing. **Daiwei Lu**: Data curation; methodology; software; writing—review and editing. **Yifan Wu**: Data curation; methodology; software. **Ipek Oguz**: Conceptualization; funding acquisition; investigation; methodology; project administration; supervision; writing—review and editing. **Nicholas Kavoussi**: Conceptualization; data curation; funding acquisition; investigation; methodology; project administration; resources; supervision; validation; writing—original draft; writing—review and editing. **Jie Ying Wu**: Conceptualization; formal analysis; funding acquisition; methodology; project administration; supervision; writing—original draft; writing—review and editing.

## CONFLICT OF INTEREST STATEMENT

The authors declare no potential conflict of interest.

## ETHICS APPROVAL

This study was performed in line with the principles of the Declaration of Helsinki. Approval was granted by the Ethics Committee of Vanderbilt University Medical Center (IRB No. 212328). Informed consent was obtained from all participants.

## FINANCIAL DISCLOSURE

None reported.

## Data Availability

The data that support the findings of this study are available on request from the corresponding author. The data are not publicly available due to privacy or ethical restrictions.

## Footnotes

1
http://www.open3d.org/docs/release/tutorial/pipelines/global_registration.html
